# Silicon–Nanodiamond-Based Anode for a Lithium-Ion Battery

**DOI:** 10.3390/nano14010043

**Published:** 2023-12-22

**Authors:** Cheng-Ying Jhan, Shi-Hong Sung, Yonhua Tzeng

**Affiliations:** Institute of Microelectronics, Department of Electrical Engineering, National Cheng Kung University, One University Road, Tainan 70101, Taiwan; m10506126@gmail.com (C.-Y.J.); william8945@gmail.com (S.-H.S.)

**Keywords:** silicon, anode, nanodiamond, battery, SEI

## Abstract

Maintaining the physical integrity of a silicon-based anode, which suffers from damage caused by severe volume changes during cycling, is a top priority in its practical applications. The performance of silicon-flake-based anodes has been significantly improved by mixing nanodiamond powders with silicon flakes for the fabrication of anodes for lithium-ion batteries (LIBs). Nanodiamonds adhere to the surfaces of silicon flakes and are distributed in the binder between flakes. A consistent and robust solid electrolyte interphase (SEI) is promoted by the aid of abundant reactive surface-linked functional groups and exposed dangling bonds of nanodiamonds, leading to enhanced physical integrity of the silicon flakes and the anode. The battery’s high-rate discharge capabilities and cycle life are thus improved. SEM, Raman spectroscopy, and XRD were applied to examine the structure and morphology of the anode. Electrochemical performance was evaluated to demonstrate a capacity retention of nearly 75% after 200 cycles, with the final specific capacity exceeding 1000 mAh/g at a test current of 4 mA/cm^2^. This is attributed to the improved stability of the solid electrolyte interphase (SEI) structure that was achieved by integrating nanodiamonds with silicon flakes in the anode, leading to enhanced cycling stability and rapid charge-discharge performance. The results from this study present an effective strategy of achieving high-cycling-performance by adding nanodiamonds to silicon-flake-based anodes.

## 1. Introduction

Lithium-ion batteries (LIBs) are one of the most important energy storage devices in modern history. They exhibit high energy efficiency and lightweight properties [1,2,3]. However, the rapid evolution of mobile electronics, electric vehicles, and high-power renewable energy technologies has caused the demand for enhanced capacity retention, energy density, lifespan, safety, and cost reduction to rise beyond the limits of state-of-the-art LIB technology [4,5,6,7].

Technological advancement in LIB anode (negative electrode) materials has focused primarily on augmenting the electrode’s capacity. Artificial graphite (AG) remains the dominant negative electrode material used in lithium-ion batteries [8]. However, current AG technology has approached a theoretical capacity limit of 372 mAh/g [9], which is insufficient for the high demands of current power cells. Hence, the development of high-capacity materials such as silicon-based negative electrodes has garnered attention.

Silicon exhibits a theoretical capacity as high as 3579 milliampere-hours per gram (mAh/g) and demonstrates an ideal lithium insertion potential (<0.5 V) [10]. Utilizing silicon as a negative electrode material shows promise for achieving energy densities surpassing 500 watt-hours per kilogram (Wh/kg) [11]. Nevertheless, practical applications of silicon electrodes face several challenges, including significant volume expansion (300–400%) and an unstable solid electrolyte interphase (SEI) [12]. The volume expansion and interphase instability of silicon–carbon anode materials have been extensively studied [13,14]. Nanoscale silicon powder reduces the effects of volume expansion. However, the high cost of nanoscale silicon particles restricts their widespread usage. High-quality silicon flake waste, a byproduct of the semiconductor industry, offers a suitable raw material for silicon anode manufacturing owing to its abundance, low cost, and recyclable nature [15]. However, the utilization of inexpensive and abundant micrometer-sized silicon flakes faces significant challenges during charge–discharge cycles owing to slow ion diffusion kinetics and notable volume expansion effects [12,16].

Several efforts have been made to suppress irreversible chemical reactions between the electrolyte and the anode, including the application of protective surface coatings such as carbon [17,18,19], lithium benzoate [20], polymers [21], and Al_2_O_3_ [22]. However, the performance of anode electrodes employing these coating materials fails to meet all of the optimal aspects of structural stability, powder filling, and conductivity required for commercial demands. Notably, in lithium-ion batteries, developing a new coating material is crucial to suppress solid electrolyte interphase (SEI) formation, maintain mechanical integrity during charge–discharge processes, and facilitate rapid electron and lithium-ion conduction. Therefore, we propose a novel strategy of incorporating nanocrystalline diamond (ND) [15] as an additive into the anode. ND exhibits excellent electrochemical and chemical inertness, along with high mechanical strength. On the other hand, the abundant dangling bonds on the surfaces of nanodiamonds allow various functional groups to attach and desirable chemical compounds to form. Other than the surfaces, most nanodiamonds possess relative chemical stability, thereby resisting erosion from various chemical processes, making it a material capable of maintaining stability in various environments [23,24,25,26].

In this study, we selected micrometer-sized silicon flakes (about 800 nm × 800 nm × 100 nm in size) as the anode material, and these flakes boast high theoretical capacity (3579 mAh/g based on Li_3.75_Si) and a flat and low charging potential (0–0.5 V vs. Li/Li^+^), rendering them promising anode candidates [27,28]. However, micrometer-sized silicon flakes have several common issues, including severe volume changes and low charging potential, leading to the decomposition of organic electrolytes and the formation of a thick solid electrolyte interphase (SEI) layer [29,30]. To demonstrate the effectiveness of ND doping, we conducted an in-depth investigation into the degradation and deterioration of original silicon anodes during cycling. The studied ND-mixed silicon anodes demonstrated their potential to exhibit highly stable long-term cycling performance and reproducibility. Therefore, we employed scanning electron microscopy (SEM), X-ray diffraction (XRD), and electrochemical measurements to study variations in electrode charge–discharge behaviors and phase transitions in the microstructure during lithiation/delithiation processes. Through these observations, we demonstrate the benefits of nanodiamonds in facilitating the formation of a uniform solid electrolyte interphase (SEI). The stable diamond-mixed uniform SEI improved high-rate performance and long-term cycling.

## 2. Materials and Methods

### 2.1. Materials

In this research, the silicon material employed was acquired from AUO Crystal Corporation, and measures approximately 800 nm in length and width, with a thickness of around 100 nm. The binder utilized was Poly (acrylic acid; PAA) provided by Eternal Materials Co. (Kaohsiung City, Taiwan), and Super P from Ubiq Technology Co. (San Diego, CA, USA) served as the conductivity enhancement additive. A copper foil with a thickness of 10 μm was selected as the current collector in the electrode fabrication process.

The nanodiamond powder was procured from Taiwan Union Abrasives Corp. (Tainan City, Taiwan). Meanwhile, the battery-grade electrolyte utilized in this investigation was obtained from Hopax Chems., MFG., Co. in Taipei, Taiwan. The electrolyte comprised a 1 M LiPF_6_ solution dissolved in equal volumes of ethylene carbonate (EC), diethyl carbonate (DEC), and dimethyl carbonate (DMC). In addition, the electrolyte contained 10 wt.% fluoroethylene carbonate (FEC).

### 2.2. Preparation of Anode

We uniformly mixed silicon flakes, binders, and conductive agent (Super P) using a weight ratio of 7:2:1. In addition, we incorporated 3 wt.% of solid 10 or 30 nm polycrystalline nanodiamond powder into the mixture. Once thoroughly mixed, the substance formed the anode slurry. Subsequently, the slurry was coated onto a copper foil with a Dr. blade and dried in a vacuum oven at 80 °C for 8 h. Finally, the samples were cut into electrodes with an area of 1.13 cm^2^.

### 2.3. Material Characterization

This investigation delved into the physical attributes and arrangement of materials through cutting-edge imaging and spectroscopic methodologies. Scanning electron microscopy (SEM) analysis was conducted utilizing a Hitachi-SU8000 instrument located in Taipei, Taiwan. Operating at an acceleration voltage of 5 kV, SEM provided comprehensive insights into the surface morphology and internal configurations of the materials.

To investigate molecular composition and vibrational characteristics, Raman spectroscopy was employed. The Raman system from Horiba Scientific (Taipei, Taiwan) utilized a green laser emitting light at 532 nm wavelength with a laser power of 450 mW, focused precisely on an area of approximately 10 µm on the sample’s surface. Through detailed analysis of Raman spectra, intricate nanostructural information on the sample was unveiled.

XRD (X-ray Diffraction) analysis, facilitated by the D8 DISCOVER with GADDS (Bruker AXS Gmbh, Karlsruhe, Germany) at a scanning rate of 4°/min, was instrumental in determining the crystallographic orientation of both the silicon powder and diamond. XRD spectra were collected on a Cu Kα beam line with a wavelength of 1.54184 Å.

In summary, these techniques were instrumental in examining and characterizing the morphology, structure, and properties of the materials under investigation.

### 2.4. Fabrication of Coin Cells

In an argon-filled glove-box environment that was meticulously controlled to maintain oxygen and moisture levels below 0.5 ppm, the assembly of electrodes into CR2032 coin cells was conducted. Lithium metal was employed as the anode material in this setup. The selected electrolyte consisted of a 1 M LiPF6 solution dissolved in a blend of ethylene carbonate (EC), diethyl carbonate (DEC), and dimethyl carbonate (DMC), each in equal proportions, resulting in a 1:1:1 volume ratio. Additionally, the electrolyte composition included the incorporation of 10 wt.% fluoroethylene carbonate (FEC).

### 2.5. Electrode Characterization

STEM and SEM were employed for the characterization of the anodes’ morphology and structures. Cycled electrodes were prepared and processed within a glove box following the procedures outlined in the references.

### 2.6. Test Cells

Galvanostatic discharge/charge experiments were conducted using a BAT battery testing system. The voltage range for the half cell spanned from 0.01 V to 1.5 V. The testing commenced after a resting period of one day. Initially, three cycles were performed at a current density of 0.2 mA/cm^2^ for the half cell, followed by subsequent cycles at 1.0 mA/cm^2^. Cyclic voltammetry (CV) and electrochemical impedance spectroscopy (EIS) measurements were conducted following the methodologies detailed in reference [31]. The CV measurements employed a scanning rate of 0.05 mV/s within a range of 0–1.5 V at room temperature. EIS measurements were recorded over a frequency range of 0.1–100 kHz.

## 3. Results and Discussion

**Figure 1:**
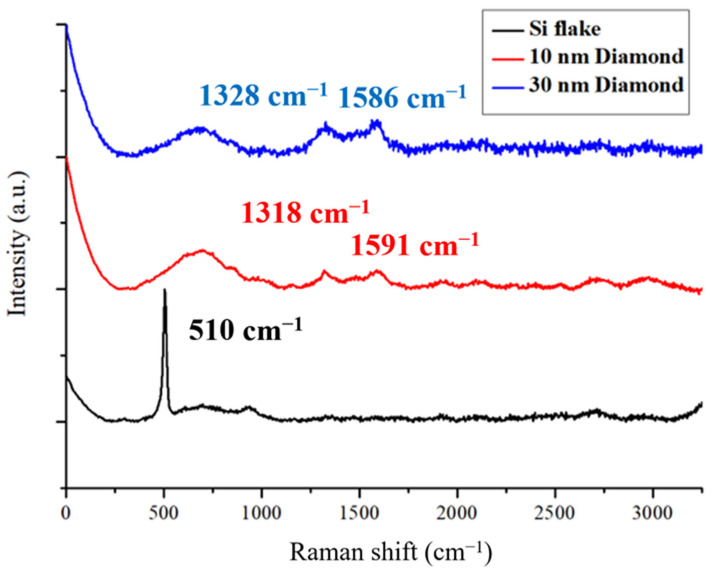
Raman spectra of Si flakes and nanocrystalline diamonds.

Figure 1 shows Raman spectra for Si-flake-based and Si-flake–nanodiamond-based Raman spectra. Nanodiamonds of 10 nm and 30 nm in sizes were used. The spectra of reference materials are primarily influenced by the first-order Raman-allowed graphite and diamond Raman scattering modes; i.e., the doubly degenerated E_2g_ at around 1586 cm⁻^1^ and the triply degenerated T_2g_ at approximately 1332 cm⁻^1^. In the case of the microcrystalline graphite sample, an additional weak feature is observed near 1430 cm⁻^1^, corresponding to the disorder-induced “D” line. This particular feature tends to be observed at lower frequencies and exhibits a significantly stronger intensity under conventional visible excitations [32,33]. The characteristic peaks of silicon crystals are clearly shown at wavenumbers of 510 and 960 cm^−1^ [34].

**Figure 2:**
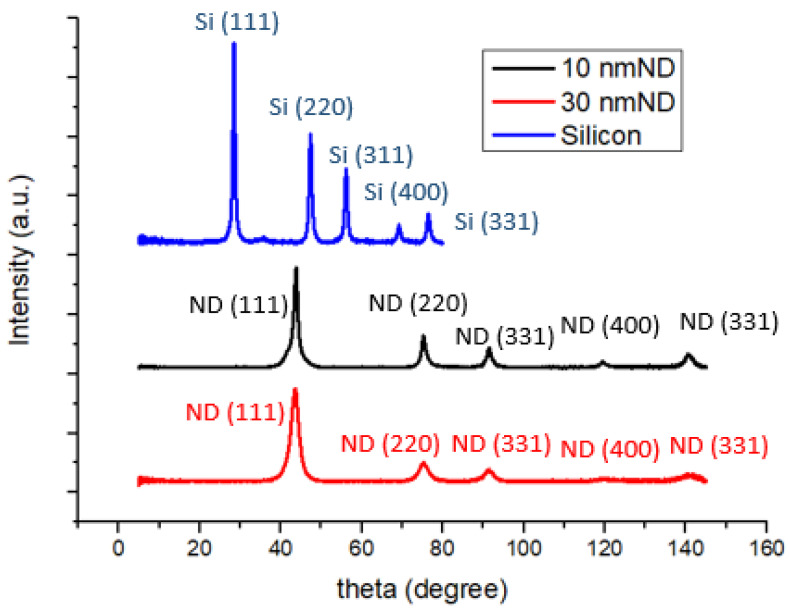
XRD patterns of anodes made of silicon and silicon–nanodiamond mixtures (10 and 30 nm).

Figure 2 illustrates the XRD spectra of silicon powder and nanodiamond powders. A relatively intense diffraction peak at approximately 2*θ* ≈ 44 degrees corresponding to the (111) lattice plane signifies that the primary crystalline phase of the diamond powder is (111), although there are orderly arrangements of other planes such as (220), (311), (400), and (331) present as well. Additionally, the diffraction characteristic peaks of nanoscale silicon powder, as analyzed previously, exhibit organized arrangements including (111), (220), (311), (400), and (331) [35,36].

**Figure 3:**
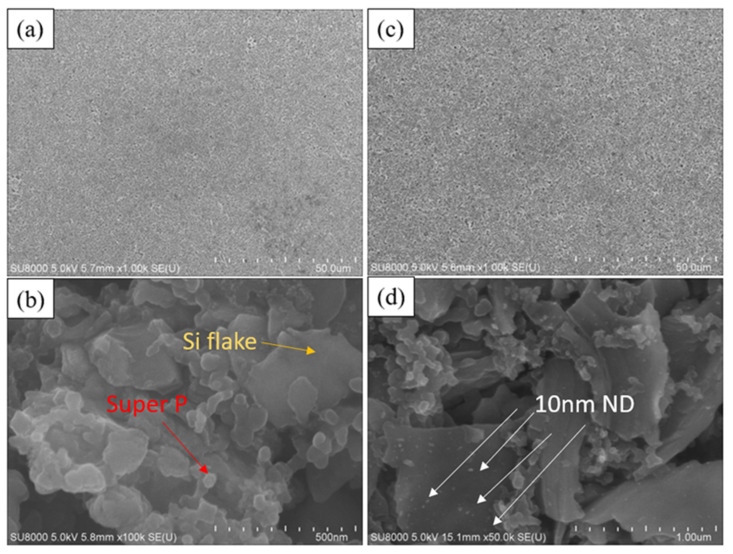
SEM image of the surface of (**a**,**b**) Si-based anode and (**c**,**d**) Si–nanodiamond-based anode.

Figure 3a,b portray the surface microstructure of a typical silicon anode, revealing irregular silicon flakes of around 800 nm (indicated by the green arrow in Figure 3b) enveloped by approximately 50 nm spherical conductive agent (Super P, indicated by the red arrow in Figure 3b). The uniform blending of silicon powder and the conductive agent form an effective conductive network. In contrast, Figure 3c,d depicts the surface microstructure of a silicon–nanodiamond anode. Unlike the conventional silicon anode, the surface of nanoscale silicon flake is coated by irregularly shaped polycrystalline diamond particles of approximately 10 nm (small dots indicated by the white arrows in Figure 3d).

**Figure 4:**
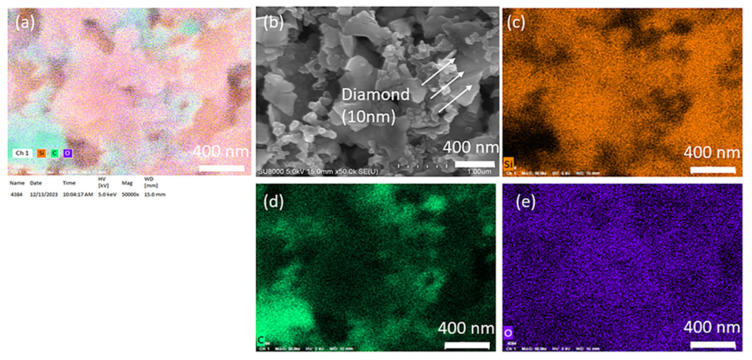
(**a**) Elemental mapping of Si-based anode with 3 wt.% 10 nm diamond, (**b**) SEM image of the surface of Si-based anode with 3 wt.% 10 nm diamond, (**c**) Si distribution, (**d**) C distribution, (**e**) O distribution of the surface of Si-based anode with 3 wt.% 10 nm diamond.

We conducted SEM mapping for C, O, and Si, as shown in Figure 4 below. Apart from diamonds, the binder also adheres to the silicon wafer surface. The PAA binder consists primarily of carbon atoms, which makes the identification of diamond by elemental analysis impractical. Directly assessing the distribution based on the size of each material in the images allows for quicker and more accurate analysis (Si flake 800 nm, Super P 50 nm, diamond 10 nm).

**Figure 5:**
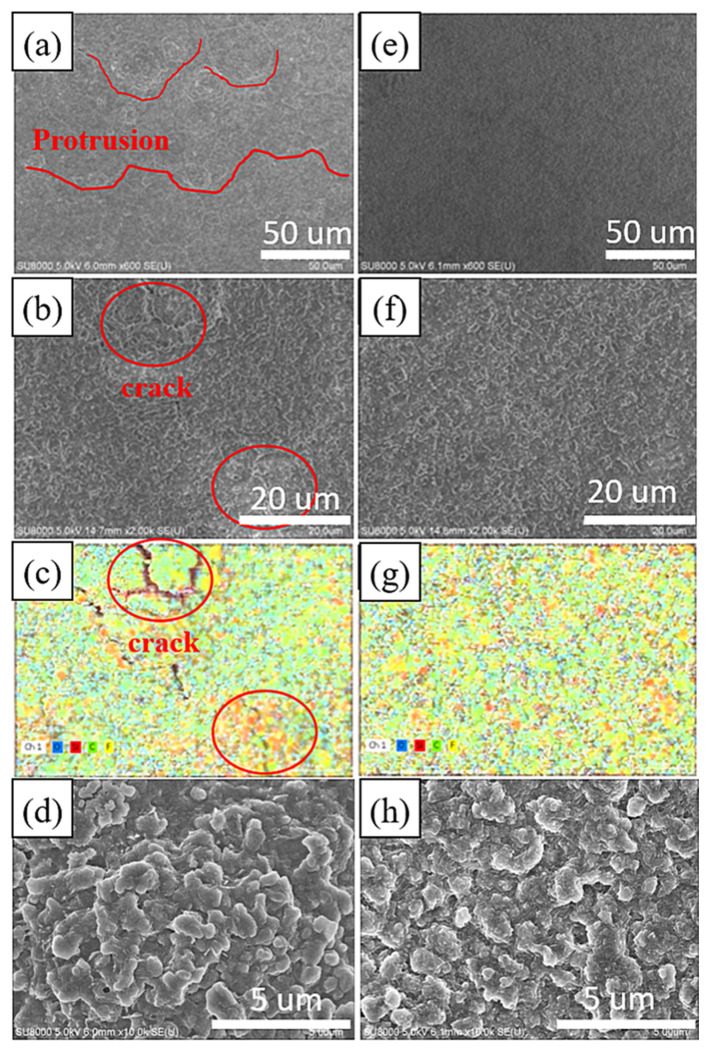
SEM image of the surface of 1st cycle Si-based anode after the 1st cycle (**a**,**b**,**d**), Si-based anode with nanocrystalline diamond (**e**,**f**,**h**) and elemental mapping of 1st cycle Si-based anode (**c**), and Si-based anode with nanocrystalline diamond (**g**).

Figure 5a–d illustrates the surface microstructure and elemental distribution of the electrode without added nanodiamond powder after the 1st cycle of discharge and charge. Oxygen is represented in blue, silicon in red, carbon in green, and fluorine in yellow. In Figure 5a, multiple reaction hotspots emerge on the electrode surface due to uneven charge and discharge, leading to non-uniform growth of the solid electrolyte interphase (SEI) layer, resulting in significant surface irregularities. The reformation of SEI in these hotspot regions perpetuates the presence of microscopic cracks and defects. These fissures or flaws facilitate direct contact between silicon particles and the electrolyte. As depicted in Figure 5c, silicon exposed within these cracks exhibits higher fluorine contents, indicating secondary reactions between bare silicon and the electrolyte. Continuous reactions between silicon-based materials and LiPF6 result in the formation of Li2SiF6 aggregates, a phenomenon previously documented by Fei Wei and colleagues [37].

Figure 5e–h depicts the surface microstructure and elemental distribution of the electrode with added nanodiamond powder after a single cycle of discharge and charge. The SEI layer on this sample’s surface appears relatively uniform and devoid of prominent reaction hotspots. The elemental distribution in Figure 5g displays a more uniform pattern, indicating more homogeneous formation of the SEI layer with enhanced mechanical strength. No localized protrusions or cracks are observed on the sample [38].

Figure 5d,h shows zoomed-in images of the microstructures corresponding to Figure 5b,f. The microstructures exhibit similar morphology, with slight differences possibly arising from the rupture of the SEI in the electrode without a nanodiamond additive.

**Figure 6:**
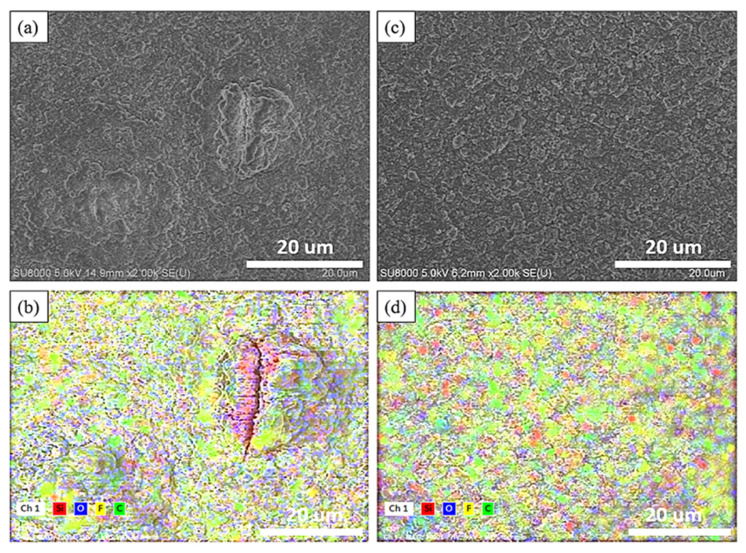
SEM image of the surface of 30th cycle Si-based anode (**a**) and Si-based anode with nanocrystalline diamond (**c**) Elemental mapping of 30th cycle Si-based anode (**b**) and Si-based anode with nanocrystalline diamond (**d**).

Figure 6a,c depicts the surface microstructure and elemental distribution of the electrode without added nanodiamond powder after undergoing 30 cycles. Oxygen, silicon, carbon, and fluorine are represented by blue, red, green, and yellow, respectively. Similar to the findings in Figure 6, the typical silicon anode surface displays protrusions larger than 10 μm after 30 cycles, indicating that silicon consequently decreased the overall electrochemical performance of the electrode. In contrast, the diamond powder-doped sample reveals a denser solid electrolyte interface (SEI) following 30 charge–discharge cycles that is free from protrusions exceeding 10 μm in size.

Following a single charge–discharge cycle, the electrode surfaces were exposed to an accelerating voltage of 5 kV for elemental composition analysis. The elemental ratios are shown in Table 1. As illustrated in Figure 6, the silicon anode without nanodiamond powder displayed a propensity for the formation of reactive hotspots, fostering thicker growth of the solid electrolyte interphase (SEI) within these areas. Originating from these focal points, micro-fractures became more prevalent, leading to silicon material exposure on the electrode surface. This increased silicon exposure readily triggered secondary reactions with the electrolyte, yielding Li_2_SiF_6_ and consequently augmenting the surface fluorine content [37].

Conversely, the electrode surface treated with supplemented nanodiamonds exhibited heightened mechanical strength and a more uniformly developed SEI. Herein, the surface silicon content measured at 1.8% was 0.42 times lower than that of the nanodiamond-free silicon anode. Additionally, the fluorine content demonstrated notably lower levels in comparison to the silicon anode devoid of nanodiamond additives. The electrode surface with fewer side reactions also exhibits lower fluorine content.

**Figure 7:**
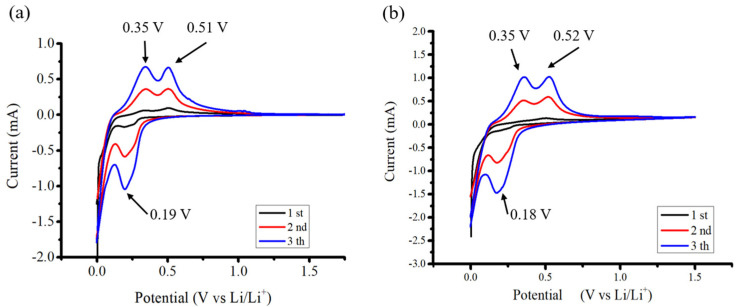
CV profiles of (**a**) Si flakes and (**b**) Si flakes with 10 nm ND.

Figure 7 shows Cyclic voltammetry (CV) experiments conducted at a scanning rate of 0.1 mVs^−1^ aimed to uncover the electrochemical characteristics of the anodes. Throughout the discharge phase, two discernible peaks emerged at 0.18 V and 0.03 V, indicating the occurrence of Si–Li alloying processes. Moreover, during the charging phase, the appearance of two peaks at 0.35 V and 0.52 V signified de-alloying processes associated with the LixSi phase. The two samples exhibit similar cyclic voltammetry curves, with the characteristic peak positions being nearly identical [39].

**Figure 8:**
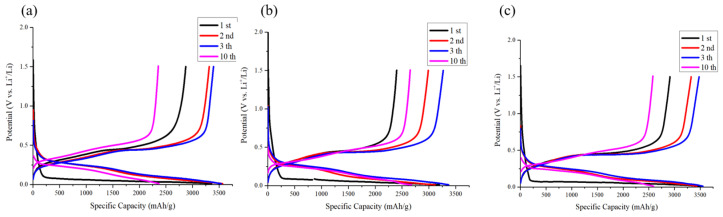
Voltage profile of anode comprising (**a**) Si flakes, (**b**) Si flakes with 3 wt.% nanodiamond (30 nm), and (**c**) Si flakes with 3 wt.% nanodiamond (10 nm).

The electrodes utilized in the study were uniformly loaded within the range of 0.9 to 1.0 mg/cm^2^. The lithium storage characteristics of these electrodes were effectively evaluated via electrochemical analysis. The initial charge/discharge curves and the first Coulombic efficiency for various electrodes at a current density of 0.2 mA cm^−2^ are illustrated in Figure 8. The Si-based anode demonstrated an initial capacity of 3565 mAh g^−1^. Notably, the Si-based anode incorporating 10 and 30 nm NDs exhibited initial capacities of 3554 mAh g^−1^ and 3409 mAh g^−1^, respectively. Moreover, the silicon anode without added nanodiamonds displayed a first-cycle Coulombic efficiency of 85.5%, whereas those integrated with 10 nm and 30 nm diamond powders exhibited Coulombic efficiencies of 83.0% and 75.1%, respectively. This notable decrease in first-cycle Coulombic efficiency is attributed to the substantial surface area of the nanodiamonds and the formation of partially irreversible compounds such as lithium carbonate on nanodiamond surfaces [40].

**Figure 9:**
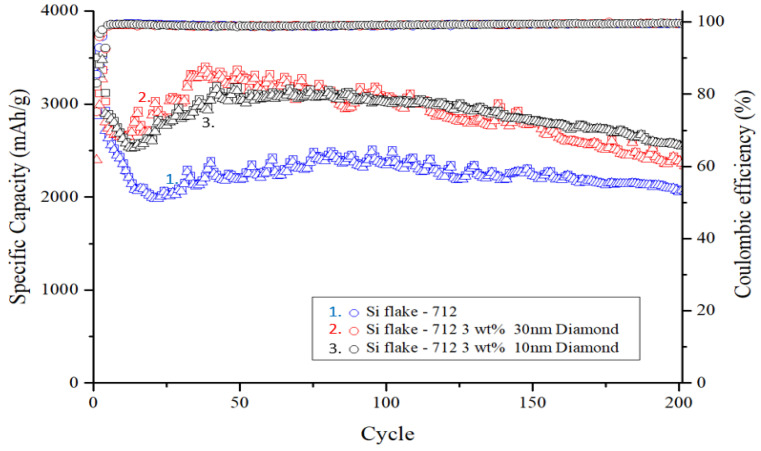
Cycle performance of silicon-based anode with and without nanodiamond.

As illustrated in Figure 9, during charge–discharge cycles, volume changes in nanosilicon particles lead to repeated rupture and reformation of the SEI layer. Silicon particles without nanodiamond additives exhibit uneven ion diffusion and often form reaction hotspots, resulting in capacity degradation. However, after 20 to 30 charge–discharge cycles, the electrode structure gradually stabilizes. Subsequently, the self-healing effect of the binder aids in gradual recovery of the electrode capacity.

The square in Figure 9. represents the discharge capacity of the anode for that cycle, while the triangle indicates the charge capacity. The Coulombic efficiency, represented by the circle, is calculated by dividing the charge capacity by the discharge capacity.

The electrode containing nanodiamond particles also displayed a trend of capacity rebound. The addition of nanodiamonds resulted in reduced decay of the initial capacity owing to the absence of reaction hotspots. This observation suggests that the surface functional groups and compounds present in nanodiamonds contribute to capacity rebound and facilitate self-healing of damage to the PAA binder, particularly amidst the significant volume expansion during silicon–lithium alloying.

Upon completion of 200 charge–discharge cycles, the silicon anode devoid of added nanodiamonds retained only 2063 mAh g^−1^, representing a capacity retention rate of 58.1% compared with its initial capacity. Conversely, silicon anodes with an added 10 nm and 30 nm of polycrystalline nanodiamond particles maintained high capacities of 2597 mAh g^−1^ and 2340 mAh g^−1^, respectively, with capacity retention rates of 74.2% and 69.5% after prolonged cycling.

**Figure 10:**
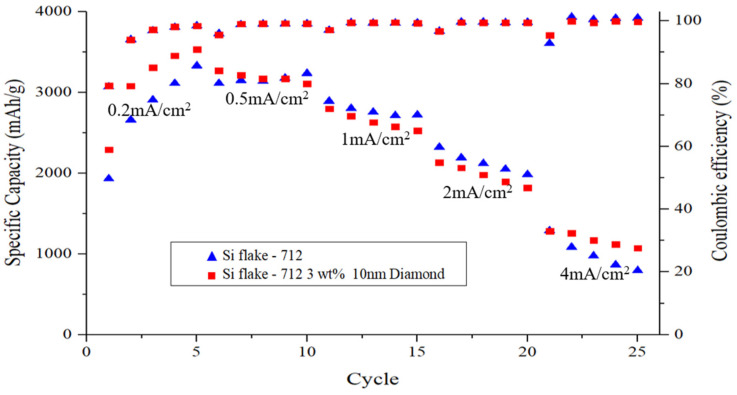
C-rate step test of silicon-based anode with or without nanodiamond.

Figure 10 illustrates the rate capability of electrodes ranging from 0.2 to 4 mA cm^−2^. Remarkably, the Si-based anode with nanodiamonds exhibited notable capacity (1010 mAh g^−1^) even at 4 mA cm^−2^, which can be attributed to its exceptional structural stability and the presence of a uniform solid electrolyte interphase (SEI) layer. Of note, from the rate-performance curves was the observation of a flatter voltage plateau for the Si-based anode with nanodiamonds, which is indicative of reduced polarization at higher test currents [41].

When silicon is mixed with diamond nanoparticles, it results in the formation of a uniform and thinner solid electrolyte interphase (SEI) layer compared with pristine silicon-based anode. This implies reduced breakdown of the electrolyte in the former scenario. Even after undergoing 200 cycles, there is no observable alteration in the morphology of the Si–nanodiamond anode, which maintains a notably thinner SEI layer, as shown in Figure 8.

**Figure 11:**
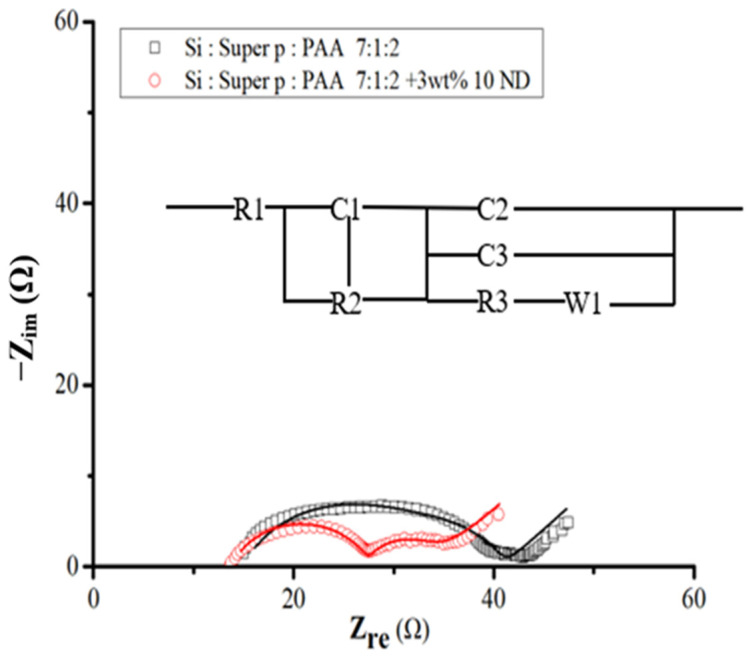
EIS spectra for Si- and Si–nanodiamond-based anodes after one cycle of discharge and charge and the equivalent circuit diagram corresponding to the EIS curves.

For a more comprehensive understanding of the system and to validate the characteristics of the SEI layer, electrochemical impedance spectroscopy (EIS) was conducted after the initial cycle. Figure 11 illustrates these spectra alongside the equivalent circuit model. Within this schematic, R1 denotes the ohmic series resistance of the cell components, R2 represents the Li ion transfer resistance through the SEI layer, and R3 signifies the charge transfer resistance. W1 pertains to the Warburg impedance linked to the diffusion of Li ions into the active electrode. The elements C1, C2, and C3 stand for constant phase elements, replacing traditional capacitor components [42,43]. Furthermore, the equivalent circuit incorporates the Li electrode component, acknowledging its influence on the overall impedance. The addition of diamond nanoparticles evidently reduces resistance, fostering an environment conducive to the alloying and de-alloying of lithium with silicon flakes.

Following the fitting process, Table 2 shows the SEI layer impedance, R2, and charge transfer impedance, R3, for Si–nanodiamond electrodes to be 14 Ω and 7 Ω respectively, contrasting with the higher values of 18 Ω and 8 Ω obtained from pristine silicon samples. The observed increase in resistance, depicted by enlargement of the semicircle in the spectra shown in Figure 11, is more pronounced in the case of pristine silicon. Moreover, the significantly lower R2 observed in Si–nanodiamond-based anode can be attributed to the presence of a uniform and thinner SEI layer, an inference drawn from electrochemical characterizations.

**Figure 12:**
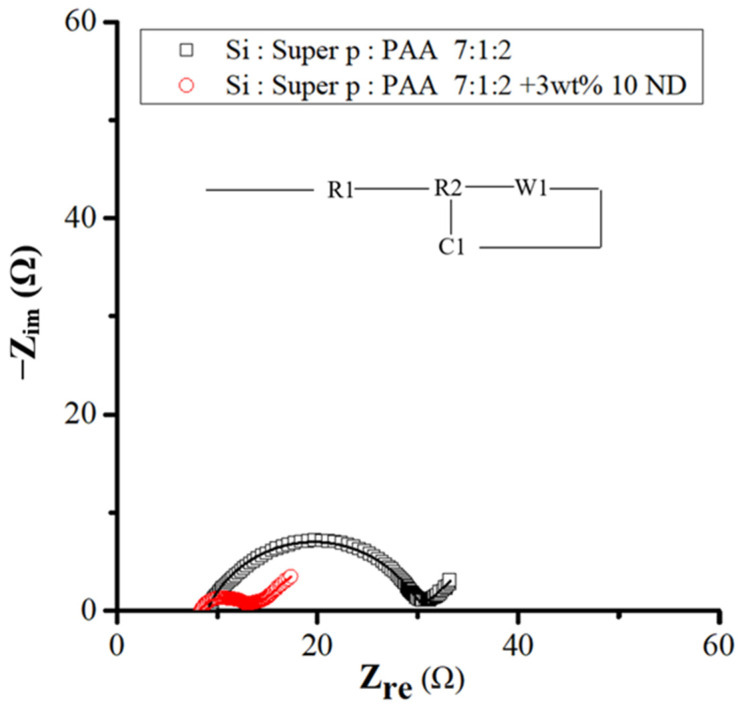
EIS spectra for Si- and Si–nanodiamond-based anodes after the 30th cycle of discharge and charge and the equivalent circuit diagram corresponding to the EIS curves.

EIS analyses were carried out on samples after 30 cycles, as shown in Figure 12, which presents Nyquist impedance plots of activated Si flakes and Si ND composite materials. Notably, both samples demonstrated a more stabilized electrode interface after the 30 cycles. Specifically, the charge transfer resistance related to the electrochemical reaction and the resistance of the SEI (solid electrolyte interphase) interface decreased to 5 ohms (R2), while the contact resistance reached 8 ohms in electrodes enriched with nanodiamond powder. These findings are shown in Table 3 and are consistent with the results of prolonged cycling studies, highlighting the positive impact of nanodiamond powder on enhancement of the stability of the solid electrolyte interphase layer.

**Figure 13:**
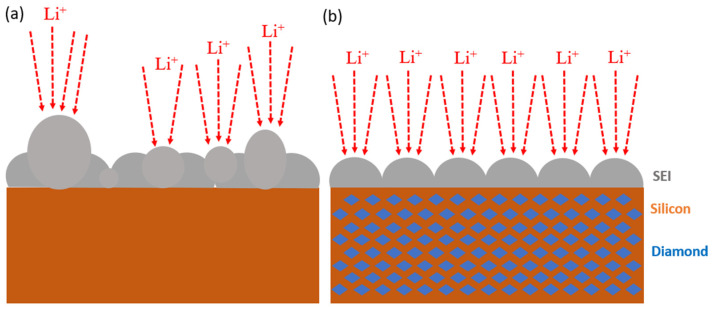
Schematic illustration of SEI layer formation in (**a**) pristine Si and (**b**) Si–nanodiamond electrodes after 1st cycle of discharge and charge.

Figure 13 illustrates the formation of the solid electrolyte interphase (SEI) layer on both silicon and silicon–nanodiamond composite electrodes. The electrode without nanodiamond tends to develop reaction hotspots, leading to the uneven growth of the local SEI layer. Areas with excessive SEI growth are prone to structural defects, resulting in the exposure of fresh silicon material. Exposed silicon further reacts with the electrolyte, thereby compromising the electrochemical performance of the electrode.

In contrast, the silicon–nanodiamond composite electrode exhibits a more uniform SEI layer, demonstrating superior mechanical properties. The surface of the composite electrode does not exhibit micrometer-scale agglomerations or cracks, indicating excellent structural stability and favorable electrochemical characteristics.

## 4. Conclusions

The performance of a silicon-based negative electrode is significantly improved by adding nanodiamonds of 10 nm or 30 nm in size to micrometer-scale silicon flake. A uniform and robust solid electrolyte interphase (SEI) layer was formed by the aid of nanodiamonds, thereby improving the mechanical stability and enhancing the high-rate discharge capabilities of the battery while extending its cycle life.

Scanning electron microscopy (SEM), Raman spectroscopy, X-ray diffraction (XRD), and electrochemical tests revealed that even after 200 charge–discharge cycles, the electrode maintained a capacity of 75% and sustained a specific capacity exceeding 1000 mAh g^−1^ under a high testing current of 4 mA cm^−2^. This underscores the continuous stability achieved in the SEI layer through the integration of nanodiamonds with silicon-based negative electrodes, resulting in improved cycling stability and advancements in rapid charge–discharge performance.

In brief, this paper presents an effective strategy involving the utilization of nanodiamond additives to a silicon-flake-based anode of a lithium-ion battery to significantly improve anode performance. Nanodiamonds are inexpensive and abundant. We believe that this paper will inspire many further research efforts in the application of nanodiamonds to achieve much higher performance in lithium-ion batteries and beyond.

## Figures and Tables

**Table 1:** Elemental composition of the surface of silicon-based anode and silicon-based anode modified by nanodiamonds.

Elemental Ratio (Atomic %)/Sample	Si	Si–Nanodiamond
C	22.7	21.4
O	68.9	75.3
F	4.17	1.51
Si	4.20	1.80

**Table 2:** Derived elemental resistance in the equivalent circuit by best fitting to the EIS curves of the pristine Si and Si–nanodiamond electrodes.

Elemental Ratio (Atomic %)/Sample	Si	Si–Nanodiamond
R1	15	14
R2	18	14
R3	8	7

**Table 3:** Derived elemental resistance in the equivalent circuits by best fitting to the EIS curves of the pristine Si and Si–nanodiamond electrodes after the 30th cycle.

Elemental Ratio (Atomic %)/Sample	Si	Si-Nanodiamond
R1	9	8
R2	21	5

## Data Availability

Data are contained within the article.
